# Undetectable mannose binding lectin is associated with HRCT proven bronchiectasis in rheumatoid arthritis (RA)

**DOI:** 10.1371/journal.pone.0215051

**Published:** 2019-04-10

**Authors:** Krista Makin, Tracie Easter, Monica Kemp, Peter Kendall, Max Bulsara, Sophie Coleman, Graeme J. Carroll

**Affiliations:** 1 Fiona Stanley Hospital, Perth, Western Australia, Australia; 2 Department of Clinical Immunology, PathWest Laboratory Medicine, Queen Elizabeth II Medical Centre, Perth, Western Australia, Australia; 3 Fremantle Hospital, Fremantle, Western Australia, Australia; 4 University of Notre Dame, Fremantle, Western Australia, Australia; Soroka University Medical Center, ISRAEL

## Abstract

**Aim:**

The aim of this study was to ascertain whether mannose binding lectin deficiency is implicated in coexistent rheumatoid arthritis and bronchiectasis and to determine whether undetectable mannose binding lectin confers poorer long-term survival in coexistent rheumatoid arthritis and bronchiectasis or in rheumatoid arthritis in general.

**Materials and methods:**

A retrospective audit was conducted in a rheumatoid arthritis cohort in which mannose binding lectin had been measured by enzyme linked immunosorbent assay from 2007–11. Rheumatoid arthritis patients with physician diagnosed HRCT proven bronchiectasis were recruited during this time and compared to those with uncomplicated rheumatoid arthritis. Survival from disease onset was recorded in October 2018. Kaplan-Meier survival estimates were performed to assess mortality over time in the two groups. Log rank tests were used for equality of survivor functions.

**Results:**

The two groups were demographically comparable. A higher frequency of undetectable mannose binding lectin was observed in coexistent rheumatoid arthritis and bronchiectasis (37.5%) compared to uncomplicated rheumatoid arthritis, (8.9%, P = 0.005). Undetectable mannose binding lectin correlated with a strong trend toward poor survival in rheumatoid arthritis overall (P = 0.057). Cox regression analysis however, showed no difference in the hazard ratio for survival between the two groups when corrected for age, gender, prednisolone use ever, rheumatoid factor status and the full range of MBL concentrations.

**Conclusion:**

In summary, undetectable mannose binding lectin is associated with coexistent rheumatoid arthritis and bronchiectasis and correlates with poor survival in rheumatoid arthritis overall. These findings further implicate immunodeficiency in the genesis of bronchiectasis in rheumatoid arthritis.

## Introduction

The association between rheumatoid arthritis (RA) and bronchiectasis (Br) is well recognised. The co-existence of these two diseases (RA+Br) is approximately 10 times that in degenerative joint disease [1]. Approximately 3% of patients with RA have clinically overt Br studies utilizing high resolution CT (HRCT) scanning of the thorax show an even higher rate of RA+Br with estimates as high as 35% [2, 3]. The importance of recognising Br and other comorbidities in RA is emphasised by the alarming differences that exist in respect to survival. For example, Swinson et al have reported that RA+Br patients are 7.3 times more likely to die during a 5-year follow-up period than the general population, 5 times more likely to die than those with RA alone and 2.4 times more likely to die than those with Br alone [4]. The aetiology of the association remains unknown. Furthermore, Puechal et al have shown poorer survival in RA+Br with a hazard ratio (HR) of 8.6 (95% CI, 1.5 to 48.2) [5].

Mannose-binding lectin (MBL) is a serum protein produced in the liver, which is an important component of the complex innate immune system. It binds to carbohydrate motifs on the surface of pathogens, damaged host cells and immune complexes. This binding allows the activation of MBL associated serine proteases (MASPs) which, when activated, allows the cleavage of C4 and C2 to form C3 convertase, thus facilitating the removal of the pathogen by direct opsonisation or by complement mediated lysis [6, 7]. By these two mechanisms, MBL is protective against various infective organisms. The concentrations of circulating MBL are determined by genetic polymorphisms in the promoter region of the MBL2 gene. Reduced concentrations (less than 1300 ng/ml) are found in people heterozygous for mutations in the MBL2 gene. Patients with severe deficiency (or undetectable MBL, less than 56 ng/ml) are likely homozygous for MBL2 mutant alleles. The frequencies of MBL variant alleles differ according to geographic location and ethnicity, but are similar in Caucasian populations [6, 8–10].

The significance of MBL deficiency in an otherwise healthy adult is unclear and still the subject of much conjecture. Several studies have concluded that MBL deficiency is only clinically relevant when there is an independent additional defect, such as neutropenia, other innate immune deficiency, iatrogenic immunosuppression or surgical stress for example. It is generally accepted that in these populations, a low serum MBL concentration may predispose to infection [11, 12]. It has also been suggested that individuals who are homozygous for the minority allele in the MBL2 gene may be at higher risk for acquiring autoimmune diseases [13]. Several recent studies have reported a significantly higher prevalence of severe MBL deficiency in patients suffering from recurrent or severe infections in RA [8, 9, 14, 15].

MBL deficiency has been linked with an increase in the frequency and severity of respiratory tract infections, particularly in RA [8, 9]. There is however, little data on the effect of MBL deficiency on the development of bronchiectasis (Br). Fevang et al found that low MBL concentrations in conjunction with common variable immune deficiency was significantly associated with an increased frequency of lower respiratory tract infections and bronchiectasis [15, 16]. In inflammatory conditions, MBL is secreted to mucosal surfaces, such as in the bronchial tracts with variable efficiency. Whether in RA in general and RA+Br in particular, there is a deficit in mucosal secretion that may contribute further to infection susceptibility, particularly in those with low or undetectable serum MBL concentrations is not yet known.

In this study, we examined the evidence for an association between MBL deficiency and the presence of Br in patients with RA. We also examined survival in RA+Br and compared it to overall survival in RA without bronchiectasis. The effect of MBL on overall survival in RA was also examined.

## Materials and methods

### Patients studied

Ethical clearance for this study, which included a waiver of consent, was obtained from the Fremantle Hospital Ethics and Human Rights Committee (2011, 233–1). A review of all patients who attended either the Fremantle Hospital Rheumatology Clinic or the private consulting rooms of one of the authors (GJC) between 1^st^ January 2007 and 31^st^ July 2011 was undertaken. Follow-up in respect to survival was then continued up until 31^st^ October 2018, when it was censored. Patients deceased thereafter were not included in the analysis. Patients with RA who had had MBL concentrations measured prior to the introduction of a synthetic disease modifying antirheumatic drug (DMARD) or a biologic agent were identified in 2011. MBL was measured in conjunction with serology for Hepatitis B and C and a QuantiFERON gold test for latent tuberculosis, as part of a strategy to minimise potential new infection or reactivated latent infection during synthetic or biologic DMARD therapy. Nineteen patients with a history of RA and clinically apparent bronchiectasis, and 124 patients with RA, but no bronchiectasis were identified.

The diagnosis of RA was reviewed and confirmed by two Rheumatologists according to the guidelines developed by the American College of Rheumatology in 1987 [17]. Amongst the 19 with RA and clinically apparent Br, 16 were found to have signs on HRCT of the chest that supported Br. Thus, the diagnosis of Br was considered HRCT proven in 16 participants. For the purpose of comparison and to maintain homogeneity, the other 3 unproven cases were removed from the analysis.

No significant changes with respect to MBL assay methodology occurred during the entire time-period over which MBL was measured in both sample groups. It is acknowledged that due to different indications and possible selection factors, the groups are not strictly comparable. Data including age, sex, diagnosis, selected disease characteristics and serum MBL concentrations was extracted from the patient’s record and MBL concentrations were corroborated against independent laboratory data.

### MBL assays

Using an automated ELISA workstation and the MBL Oligomer ELISA kit (Bioporto, Hellerup, Denmark), the concentration of oligomerised MBL in human serum was measured as per the manufacturer’s instructions.

Briefly, the 4 step procedure is as follows:

1. Diluted patient serum is added to micro wells pre-coated with a monoclonal antibody against the MBL carbohydrate-binding domain.
2. Biotinylated MBL antibody is then added which binds to any carbohydrate domains not already bound down to the micro well coat.
3. Horseradish peroxidase conjugated streptavidin is then added to form a complex with the bound antibody
4. Tetramethylbenzidine substrate is finally added to generate a coloured product.

Washing is performed between steps to remove unbound material. The colour reaction is stopped and the colour intensity read at 450nm. The intensity of the resulting coloured product is directly proportional to the concentration of MBL in the serum.

The measuring range for the assay is from 0 to 4000ng/mL. The testing laboratory uses a threshold of 56ng/mL based on a historical cut-off due to the standards originally used. As some of the samples used in this study were measured at this initial time, we maintained this threshold for all samples. We acknowledge that the assay can measure lower values and the currently most used cut-off in the literature is 50 ng/mL [9].

### Statistical analysis

The significance of differences in respect to demographic data, such as age and gender distribution was determined using unpaired t tests in respect to the former and 2x2 contingency tables and Chi-squared tests in respect to the latter. Frequencies of MBL deficiency within each group were compared using the Fisher’s exact test. Kaplan-Meier survival estimates were performed to assess mortality over time in the two groups and to compare survival according to MBL status. Log rank tests were used for equality of survivor functions. Cox proportional hazard regression model was used to compare mortality rates controlling for confounders. P values < 0.05 were considered statistically significant.

## Results

The demographic features and selected disease characteristics of participants are shown in Table 1. Although differences in age, gender distribution and corticosteroid use between the participants in the RA+Br group and the RA without bronchiectasis group were observed, these findings were not statistically significant and are of doubtful relevance. In all other respects the two groups were comparable.

**Table 1:** Demographics and disease characteristics. The significance of differences was determined by Fisher’s exact test for categorical variables* and by unpaired t tests for continuous variables^#^.

	RA and Br	RA without Br	Significance of differences
Number	16	124	
Mean age (years)	73.2	67.1	^#^P = 0.07
Gender (female)	13 (81%)	86 (69%)	*P = 0.395
Ethnicity (Caucasian)	15 (94%)	120 (97%)	*P = 0.46
RA disease duration (years)	17.6	14.5	^#^P = 0.295
RF status (positive)	11 (79%)	86 (74%)	*P = 1.00
Ever smoker	6 (46%)	42 (39%)	*P = 0.766
Prednisolone ever	9 (56%)	87 (70%)	*P = 0.265

Anti-CCP antibody was measured in 7 of the 16 patients (43%) of the RA+Br group and in 27 of the 124 patients (22%) of the RA without bronchiectasis comparator group. Six out of 7 (86%) patients in the RA+Br group were positive for anti-CCP antibody compared to 17 out of 27 in the RA without bronchiectasis group. This difference in frequency was not statistically significant (P = 0.5).

The use of medical therapies including corticosteroids, conventional synthetic DMARDs and biologic DMARDs is documented in Table 2.

**Table 2:** Different frequencies of usage of csDMARDs and bDMARDs in RA+Br and RA without bronchiectasis.

	RA+Br	RA controls	Sig of Differences
No. (%) who ever had oral CS	10 (63%)	85 (69%)	NS**
Median number of csDMARDs used (95% CI)	2 (1.40–2.85)	3 (2.43–2.86)	P = 0.0432*
No. in whom bDMARDs were used	4 of 16 (25%)	86 of 115 (75%)	P = 0.0002**

CS = corticosteroid therapy; csDMARDs = conventional synthetic disease modifying anti-rheumatic drugs; bDMARDs = biologic DMARDs

*denotes unpaired t-test

**denotes Fishers exact test.

In the 16 patients with RA+Br, the median number of lung lobes affected was 2, range 1 to 5. Multi-lobar disease (HRCT determined involvement of 2 or more lobes) was observed in 13 of the 16 (81%) participants. A weak correlation was noted between the number of affected lobes and the MBL concentration (Pearson correlation coefficient, R = 0.2251, P = 0.4199). In 25 of the 124 RA without bronchiectasis group, respiratory investigations including chest x-ray, pulmonary function tests and HRCT were performed where there was clinical suspicion of pulmonary pathology. Interstitial lung disease was present in 11 patients (8.9%), chronic obstructive pulmonary disease was present in 10 patients (8.1%), lung cancer was found in 2 patients (1.6%), pulmonary tuberculosis and bronchiolitis were present in 1 patient each (0.8%). Overall 20.2% of the RA without bronchiectasis group were found to have pulmonary pathology other than bronchiectasis.

**Fig 1:**
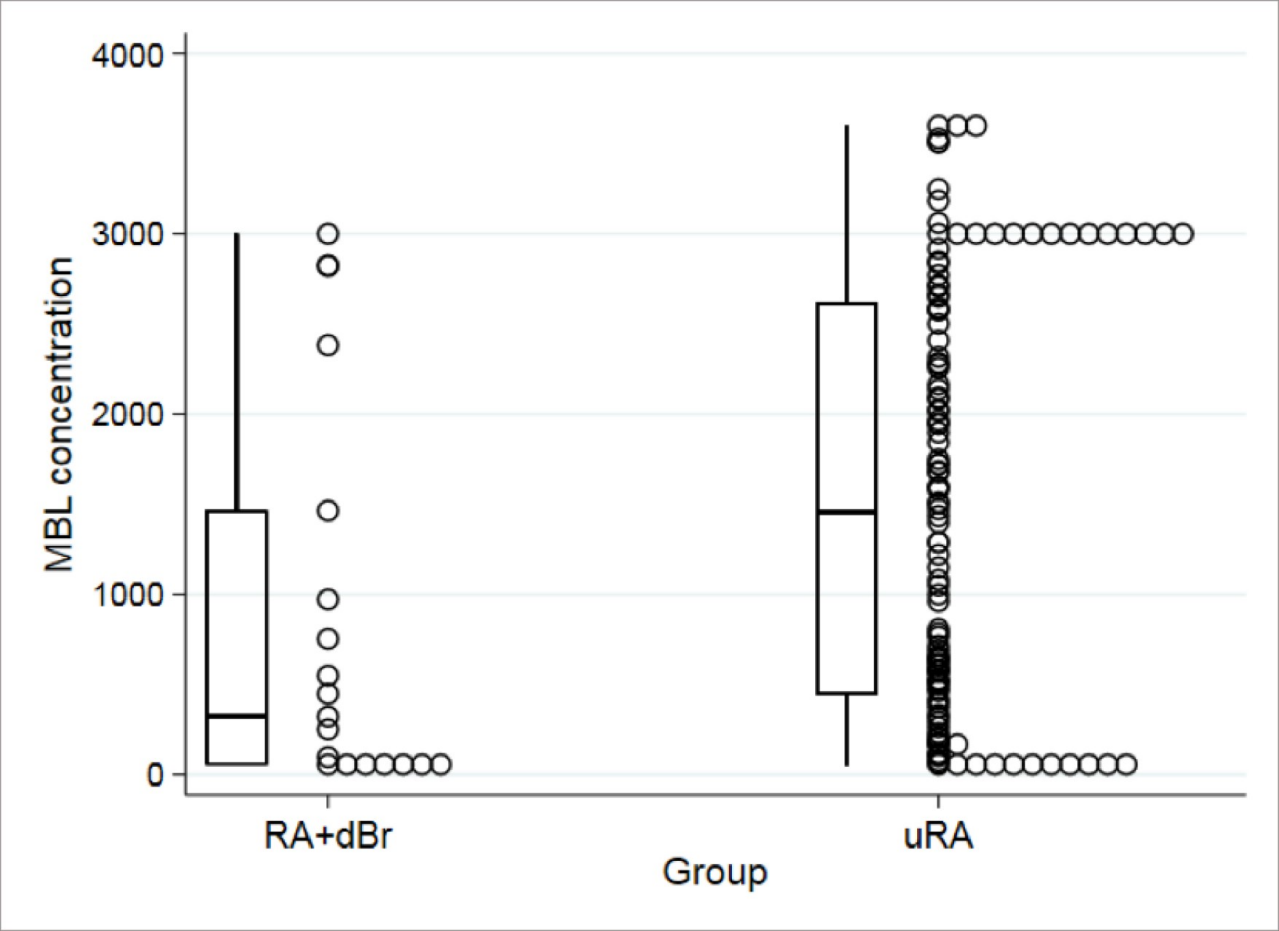
Distribution of MBL concentrations in RA+Br and in the comparator group (RA without bronchiectasis). Boxplots denote the median values (bars) and the 25^th^ and 75^th^ centiles at each end of the boxes, while whiskers denote the extremes of the range.

The distribution of MBL concentrations in the RA+Br and the RA without bronchiectasis groups is shown in Fig 1.

A clear difference in undetectable MBL (uMBL) concentrations was observed in the RA+Br group compared to the RA without bronchiectasis group, as can be seen by the proportion of uMBL concentrations in the dot plot.

A considerably higher frequency of undetectable MBL was noted in the RA+Br group (37.5%) compared to RA without bronchiectasis group (8.9%, P = 0.005).

**Fig 2:**
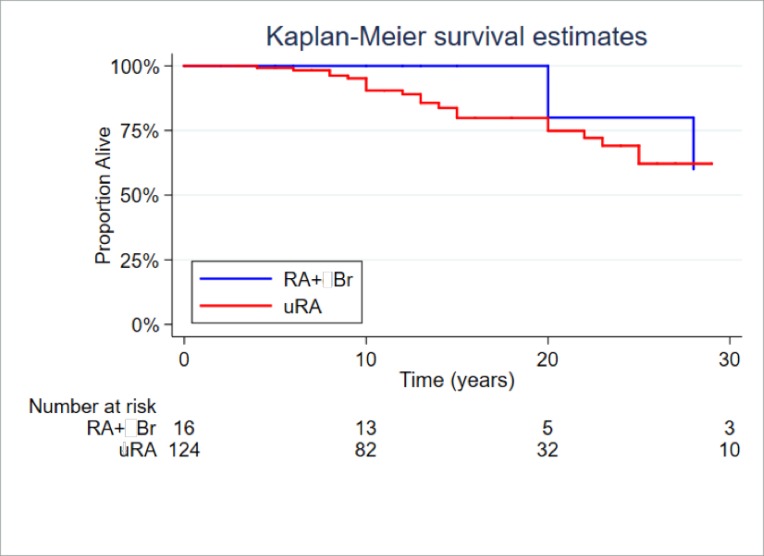
Kaplan-Meier survival estimates for coexistent RA+Br versus RA without bronchiectasis.

Slightly poorer survival was observed in the RA+Br group (38%) compared to RA without bronchiectasis (19%) as can be seen in Fig 2, but when Kaplan-Meier survival curves were compared, our findings were not as impressive as those of Puechal et al [5]. In our study the small observed difference was not statistically significant, possibly due to the relatively small number of RA+Br participants.

Cox regression analysis was employed to evaluate the frequency of deaths in the two groups in this study. The frequency of deaths does not differ significantly (38% in RA+Br versus 19% in RA without bronchiectasis, Log-rank test for equality of survivor functions P = 0.71). The difference between groups was not statistically significant after controlling for age, gender, corticosteroid use, rheumatoid factor and MBL concentrations (HR = 1.74; (95% CI 0.33 to 9.19); P = 0.51).

**Fig 3:**
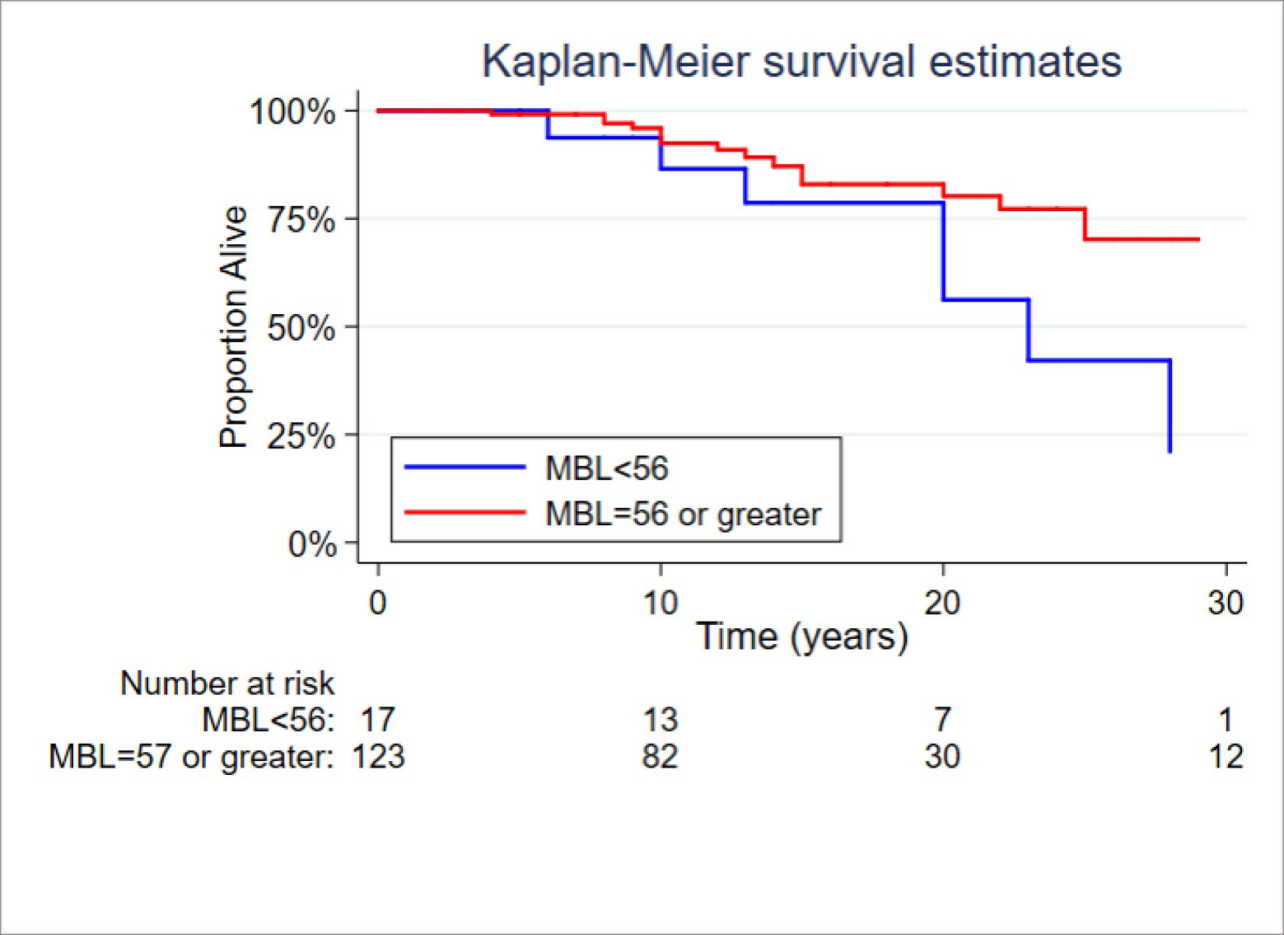
Kaplan–Meier survival curves indicating survival according to MBL status.

A Kaplan–Meier survival curve demonstrating survival according to MBL status is shown in Fig 3. In this study, undetectable MBL correlated with poor survival (P = 0.057) in RA overall.

We did not compare the RA+Br and the RA without bronchiectasis groups in respect to comorbidities, however we did not note any obvious imbalance in relevant comorbidities, such as asthma, COPD, cystic fibrosis, lymphopaenia or hypogammaglobulinaemia, for example. Participants had total lymphocyte counts and serum immunoglobulins measured at the same time as the MBL. For lymphocyte counts, mean = 1.63 in the RA+Br group and 1.92 in the RA without bronchiectasis group, P = 0.280 and for serum immunoglobulins, mean IgG = 9.79 in the RA+Br group and 9.78 in the RA without bronchiectasis group, P = 0.9968). Thus, no major differences between groups were observed in respect to these markers of immunodeficiency.

## Discussion

In this retrospective survey of patients with coexistent RA and Bronchiectasis (RA+Br) and RA without bronchiectasis, a much higher frequency of undetectable MBL was observed in RA+Br (38%) compared to RA without bronchiectasis (9%). The frequency of undetectable MBL in RA without bronchiectasis accords with that reported in other Caucasian RA populations [6, 10]. These findings suggest a possible causative link between MBL deficiency and the development of bronchiectasis in the RA population.

Many of the study subjects had longstanding RA and the diagnosis was not in doubt. In most study participants anti-CCP antibody test was not perceived necessary to confirm the RA diagnosis and thus only a small number of participants in either group had an anti-CCP antibody performed. In those subjects who did have the test performed there was an increased frequency in the RA+Br group. This difference in frequency was not statistically significant, but this may be attributable to the relatively small numbers. The reported frequency of anti-CCP antibody in RA is between 55% and 69% [18, 19]. Perry et al in an observational study of 53 patients with both RA and bronchiectasis reported a significantly higher frequency of anti-CCP antibody in the RA+Br group (83%) compared with a matched RA without bronchiectasis group (46%) (P < 0.001) [20]. Our results are in keeping with these findings.

Both conventional synthetic DMARDs and biologic DMARDs were used less frequently in the RA+Br group. It is possible that these agents were avoided due to fear of exacerbations of bronchiectasis, or fear of potentially serious infections in the context of immunosuppression. Corticosteroids may have been avoided in subjects with RA+Br for the same reason. We consider it unlikely that disease-modifying therapies contributed to or altered the pathogenesis of bronchiectasis in this cohort, however we cannot exclude such a contribution with certainty.

A high frequency of biologic DMARD use was observed amongst RA without bronchiectasis group (Table 2). Most subjects in this cohort underwent MBL testing as a pre-biologic screen at a time when treatment escalation was being considered. It follows that since bDMARDs were often about to be commenced, the RA without bronchiectasis cohort may represent more severe RA and as such may not be well matched to the RA+Br cohort. Given the observational nature of this study it was not possible to better match the control group.

Bronchiectasis is strongly associated with RA. The frequency of bronchiectasis in patients with RA is approximately 3%, which is about 10 times that in a sample of patients with osteoarthritis (OA) derived from a similar population [1]. Investigators in the UK have reported that Br mostly precedes the development of RA [1], whereas those in the USA have reported that Br mostly occurs after the development of RA [21, 22]. The reason for the strong association between RA and Br is not known and is still the subject of investigation. Several possible explanations have been postulated. Chronic suppurative infection may predispose to the development of RA due to persistent antigenic stimulation and the possible development of an auto-immune response that may involve molecular mimicry between a bacterial surface component for example, such as a peptidoglycan and a molecular constituent within joint tissues. Alternatively, some patients destined to develop RA may not be able to clear infections due to single or multiple defects in their innate immune system, such as MBL deficiency, complement component deficiencies CVID or selective T or B cell deficiencies. Such a predilection may be compounded by exposure to glucocorticoids, synthetic DMARDS or biologic DMARD therapies, alone or in combination. Furthermore, the presence of secondary Sjogren’s syndrome in RA may be contributory, due, for example, to depressed production of mucous in the airways and possibly other mechanisms that favour persistent airway infection. Whether MBL deficiency is more common in RA patients who develop secondary Sjogren’s syndrome is unknown. There is no evidence to suggest that MBL deficiency has any influence on the development of Sjogren’s syndrome. Rather, recent data suggests that the presence of an MBL deficient genotype may be protective against the development of aggressive auto-immune damage in patients suffering from the primary form of this disease [23].

In a novel, family-based association study, Puechal et al have observed a strong association and linkage between heterozygous mutations in the cystic fibrosis transmembrane regulator (CFTR) gene in patients who have both RA and Br [5]. They suggest that CFTR mutations may be an important risk factor for the development of Br in patients with RA [24]. It would be interesting to determine whether the frequency of and survival in RA+Br is different in those with combined CFTR heterozygosity and undetectable MBL compared to those with neither.

It is generally accepted that MBL deficiency does not play a key role in rheumatoid disease pathogenesis. Previous studies have not revealed any difference in the frequencies for MBL deficiency in RA and healthy population controls [16]. Low MBL concentrations however, may affect RA disease progression. In an early RA study, Saevarsdottir et al found that MBL deficiency was a predictor of poor outcome characterized by erosive joint disease as determined by plain X-rays, reduced responsiveness to conventional synthetic DMARD therapy and higher concentrations of rheumatoid factor [10]. Accordingly, it may be argued that MBL deficiency leads to more severe RA, which in turn is associated with more intense and sustained inflammation and probably worse survival, for which our data lends some support. In more severe RA, there is a need for greater pharmacological intervention, such as the use of oral and intra-articular glucocorticoids more often and at higher average doses, together with the use of more synthetic DMARDs at higher doses and/or in combination. Moreover, it is these patients who are more likely to end up qualifying for and being treated with biologic DMARDs, albeit at potentially still greater risk for infection, at least initially over the first 1–2 years of biologic therapy. Singularly or in combination, these factors may increase patient susceptibility to Br. Although plausible, we consider this explanation for the association of RA and Br to be improbable.

Alternatively, and in our opinion, more likely, is the possibility that in RA, MBL deficiency is itself an important risk factor for lower respiratory tract infection and in turn a risk factor for promoting or compounding Br. Undetectable MBL may be indicative of, or at least a marker for immunodeficiency syndromes in RA, which in turn predispose to sino-pulmonary infections as is the case for example in common variable immune deficiency (CVID) in association with RA and possibly also coexistent RA and yellow nail syndrome, in which the frequency of Br is reported to be up to 89% [25]. Furthermore, MBL deficiency may be a generic risk factor for many types of suppurative and other infections in RA. The findings of Nisihara et al and the recently published findings in a separate RA cohort support this proposition [8, 9]. In the Brazilian cohort, MBL deficiency was associated with statistically significantly higher frequencies of bronchitis and a trend toward increased frequency of pneumonia in RA. That lack of MBL availability at the airway surface could be relevant to the genesis of Br in RA is supported by the data of Fidler et al, who argue for a role for MBL in pulmonary defence, based on the findings in a bronchoalveolar lavage study of MBL concentrations in children with acute and chronic airway infections [26].

Outcome in RA is poorer than in population controls and in this and other studies it appears to be poorer still amongst the subset of patients with RA and concomitant bronchiectasis, in large part due to an increased frequency of pulmonary infection [26]. Worse survival has been demonstrated in several other studies and although not statistically significant in this study, a very similar trend and Kaplan-Meier survival plot was observed [4, 5, 24]. Puechal et al showed poorer survival in RA+Br compared to RA without bronchiectasis, but their study included a larger number of RA+Br participants (n = 30) and a smaller number of RA without bronchiectasis participants (n = 25). It must be admitted that neither study was well powered in view of the relative rarity of RA+Br in most centres.

Strengths of this study include the HRCT confirmation of Br in all 16 RA+Br participants (100%), the relatively long duration of RA in all participants, which limits the likelihood that Br in those destined to develop the condition might not yet have manifested and the long-term follow up, which permits potentially more accurate determination of long-term survival outcomes. In addition, it is considered that the robust independently validated MBL assay methodology, upon which we relied, represents a further important strength.

The limitations of this study include the absence of a healthy population control group, and a non-RA Br control group, the relatively small numbers in the RA+Br group, which limits statistical power, and the potential for unequal matching between groups for factors such as tobacco use, which was not systematically determined, as well as atopy and exposure to environmental and occupational respiratory irritants. Ever smokers were similar in the two groups, but no further details concerning cumulative tobacco use were available. Other shortcomings include the limited data available concerning therapies, the incomplete details concerning glucocorticoid exposure (only prednisolone ever was available, not maximum dose, average dose over time or cumulative dose) and the lack of details regarding precisely which synthetic and biologic DMARDS had been used and in what dosages and combinations and for how long. Moreover, limited information was available concerning the presence or absence of other disorders that may contribute to deficient innate or acquired immunity.

## Conclusion

In conclusion, the findings demonstrate a statistically significant association between undetectable MBL and coexistent RA and bronchiectasis. Poorer survival was observed in RA+Br compared to RA without bronchiectasis as in other studies, however this was not statistically significant in this study, possibly due to relatively small numbers. A strong trend toward poorer survival in all RA participants with undetectable MBL was noted (P = 0.058). A larger cohort study is required to further evaluate this trend and investigate whether the increased mortality can be accounted for on the basis of pulmonary infection, infections in general or other factors, such as cardiovascular events or more severe RA. Undetectable MBL may be a risk factor for the development of Br in RA and may have aetiopathogenetic importance as well as prognostic significance in respect to worse survival in RA overall. Further studies of a more rigorous and preferably prospective nature need to be undertaken to confirm these preliminary observations and importantly, to elucidate the pathogenetic role of MBL in RA+Br as well as in respect to the generic risk for pulmonary and other infections in RA. Closer examination of immunodeficiency phenomena, which can co-exist with RA, such as MBL deficiency alone and MBL deficiency in association with other immune defects, including hypogammaglobulinaemia as well as innate T and B cell disorders, may shed new light on the interesting interface between immunodeficiency and auto-immunity. Moreover, such scrutiny may provide greater understanding of the immunopathogenesis of both conditions. Treating clinicians currently face much uncertainty as to how best to safely and yet efficaciously treat patients with RA+Br, who are at greater risk for serious infection and more so still, should therapy lead to additional immunocompromise. Knowledge of their innate immunocompetency including MBL status may inform decision-making in respect to therapy and heighten clinician vigilance in respect to infection, which in turn should improve patient outcomes.

## Supporting information

S1 File Data-set. Anonymized RA+Br and uRA data 20190318.
(XLSX)

## Abbreviations

MBL: Mannose binding lectin
Br: Bronchiectasis
RA: Rheumatoid arthritis
HRCT: High resolution CT scan
HR: Hazard ratio
DMARD: Disease modifying antirheumatic drug
CFTR: Cystic fibrosis transmembrane conductance regulator
COPD: Chronic obstructive pulmonary disease
